# Ribosome Shunting, Polycistronic Translation, and Evasion of Antiviral Defenses in Plant Pararetroviruses and Beyond

**DOI:** 10.3389/fmicb.2018.00644

**Published:** 2018-04-10

**Authors:** Mikhail M. Pooggin, Lyubov A. Ryabova

**Affiliations:** ^1^INRA, UMR Biologie et Génétique des Interactions Plante-Parasite, Montpellier, France; ^2^Institut de Biologie Moléculaire des Plantes, Centre National de la Recherche Scientifique, UPR 2357, Université de Strasbourg, Strasbourg, France

**Keywords:** ribosome shunting, leaky scanning, reinitiation, upstream ORF, secondary structure, RNA interference, innate immunity, autophagy

## Abstract

Viruses have compact genomes and usually translate more than one protein from polycistronic RNAs using leaky scanning, frameshifting, stop codon suppression or reinitiation mechanisms. Viral (pre-)genomic RNAs often contain long 5′-leader sequences with short upstream open reading frames (uORFs) and secondary structure elements, which control both translation initiation and replication. In plants, viral RNA and DNA are targeted by RNA interference (RNAi) generating small RNAs that silence viral gene expression, while viral proteins are recognized by innate immunity and autophagy that restrict viral infection. In this review we focus on plant pararetroviruses of the family *Caulimoviridae* and describe the mechanisms of uORF- and secondary structure-driven ribosome shunting, leaky scanning and reinitiation after translation of short and long uORFs. We discuss conservation of these mechanisms in different genera of *Caulimoviridae*, including host genome-integrated endogenous viral elements, as well as in other viral families, and highlight a multipurpose use of the highly-structured leader sequence of plant pararetroviruses in regulation of translation, splicing, packaging, and reverse transcription of pregenomic RNA (pgRNA), and in evasion of RNAi. Furthermore, we illustrate how targeting of several host factors by a pararetroviral effector protein can lead to transactivation of viral polycistronic translation and concomitant suppression of antiviral defenses. Thus, activation of the plant protein kinase target of rapamycin (TOR) by the *Cauliflower mosaic virus* transactivator/viroplasmin (TAV) promotes reinitiation of translation after long ORFs on viral pgRNA and blocks antiviral autophagy and innate immunity responses, while interaction of TAV with the plant RNAi machinery interferes with antiviral silencing.

## Introduction

Viruses tend to evolve compact genomes with closely spaced or overlapping protein-coding sequences and rather short non-coding sequences stuffed with multiple regulatory *cis*-acting elements. This compactness allows for more efficient replication and encapsidation as well as successful competition with host mRNAs for translation in the cytoplasm and, in the case of DNA viruses, with host chromosomes for transcription and replication in the nucleus. Retroviruses and pararetroviruses that visit both cytoplasm and nucleus during their replication cycles are among the best representatives of such compactness and multipurpose use of regulatory regions of a viral genome (reviewed in Rothnie et al., 1994; Ryabova et al., 2002). Unlike retroviruses, pararetroviruses do not have an obligatory host genome integration step, encapsidate into virions a circular dsDNA genome, and replicate via reverse transcription of pgRNA. The pgRNA is transcribed by Pol II from the circular dsDNA episomes accumulating in the nucleus and then transported to the cytoplasm for translation and eventually reverse transcription.

In this review we focus on plant pararetroviruses (family *Caulimoviridae*) that have evolved (i) short upstream (u)ORF-dependent ribosome shunting to regulate sorting of pregenomic RNA (pgRNA) for translation and reverse transcription, and (ii) leaky scanning and virus-activated reinitiation to make ribosomes translate two and more long ORFs from one polycistronic RNA. Using as examples the best studied pararetroviruses *Cauliflower mosaic virus* (CaMV; genus *Caulimovirus*) and *Rice tungro bacilliform virus* (RTBV; genus *Tungrovirus*), we highlight a multipurpose use of their pgRNA leader sequence in regulation of ribosome shunting, RNA splicing, packaging and reverse transcription as well as in evasion of RNAi-based antiviral defense. We also describe an uORF- and secondary structure-dependent ribosome shunt mechanism evolved by other viral families such as animal (para-)retroviruses (families *Hepadnaviridae* and *Retroviridae*) and plant RNA picorna-like viruses (family *Secoviridae*). We then describe the mechanisms of polycistronic translation of pgRNA in CaMV and RTBV and discuss the conservation of these mechanisms in different genera of *Caulimoviridae*, including host genome-integrated endogenous viral elements. Specifically we focus on the mechanism of reinitiation after long ORF translation, transactivated by the CaMV TAV protein through its interaction with several components of the cellular translation machinery and activation of the protein kinase TOR. Finally we describe the mechanisms of CaMV TAV-mediated suppression of plant antiviral defenses based on RNAi, innate immunity and autophagy, and discuss a possible role of the RTBV protein P4 in suppression of RNAi and innate immunity.

## The Regulatory Role of Short uORFs in 5′-Leader Regions of Eukaryotic mRNAs

In eukaryotes, translation is usually initiated via a cap-dependent scanning mechanism (Kozak, 1999, 2002). According to a current model, the 43S preinitiation scanning complex composed of the 40S ribosomal subunit, a ternary complex (eIF2/GTP/Met-tRNAi^Met^) and several translation initiation factors (eIF1, eIF1A, eIF3, and eIF5), loaded at the capped 5′-end of mRNA via the multisubunit cap-binding complex eIF4F (eIF4E/eIF4G/eIF4A/eIF4B), scans the mRNA leader sequence linearly until the first AUG triplet in a favorable initiation context is encountered, where the 60S ribosomal subunit joins to form the 80S ribosome and elongation begins (reviewed in Jackson et al., 2010; Browning and Bailey-Serres, 2015). The favorable initiation context usually contains both R (A or G) at position -3 and G at position +4 with respect of the first nucleotide (nt) of the start codon (Kozak, 1986). A substantial fraction of the scanning ribosomes can bypass the first AUG and reach a downstream start codon in a process of leaky scanning if the first AUG resides in a moderate context missing either R or G at the corresponding positions, or if it is located too close (less than 10 nts) to the 5′-cap (Kozak, 1991). Only a small fraction of the scanning ribosomes can recognize AUGs residing in a weak context missing both favorable nts at the positions -3 and +4. In the cases of weak and moderate contexts of AUG, a secondary structure positioned at a short distance of ca. 14 nts downstream of the suboptimal start codon can strongly increase efficiency of translation initiation, likely by retarding the scanning ribosome and thereby providing more time for start codon recognition (Kozak, 1990). Notably, translation can be initiated with low efficiency even at non-AUG start codons which deviate from AUG at one position (e.g., CUG, AUU, etc.) (Gordon et al., 1992), and such initiation is facilitated by a favorable initiation context, downstream secondary structure, and/or other factors (discussed below). After termination of translation, the 80S ribosome disassembles and usually cannot reinitiate translation at a downstream ORF on the same mRNA, except when the upstream ORF is shorter than about 50 codons (Fütterer and Hohn, 1992; Kozak, 2001): in the latter case the released 40S ribosomal subunit appears to be able to reacquire the ternary complex and the 60S ribosomal subunit *de novo* to initiate at the next start codon. The translation initiation factors (eIFs) acquired during the cap-dependent translation initiation event are thought to dissociate from the translating ribosome gradually during the first few elongation cycles. If the translation event is short, eIFs can still be available for downstream reinitiation. In contrast, eIFs appear to be completely lost from the translating ribosome during multiple elongation cycles and become unavailable for a downstream reinitiation event after long ORF translation, unless a special viral reinitiation factor keeps them attached to the translating ribosome (discussed below).

Bioinformatic analyses have revealed a large subset of eukaryotic mRNAs that contain in the 5′-leader region one or more short uORFs, which can potentially be recognized by scanning ribosomes and translated (Tran et al., 2008; von Arnim et al., 2014; Johnstone et al., 2016). Likewise, 5′-leaders of viral RNAs often contain regulatory short uORFs. Generally, uORFs are considered to be repressors of downstream translation. Notably, some short uORFs such as conserved peptide uORFs (CPuORFs) encode attenuator peptides which act in a sequence-dependent manner to inhibit their own translation termination and thereby repress translation of the main ORF (reviewed in von Arnim et al., 2014). However, the coding content, the stop codon context and/or the position of a short uORF with respect of the main ORF or other uORFs in the leader sequence can influence its effect on downstream translation. Contrasting effects of short uORFs on downstream translation can be exemplified by the case of GCN4 translation control in yeast, where the 5′-proximal uORF translation allows the post-terminating ribosomes to resume scanning, conditionally bypass the 3′-proximal inhibitory uORF4 and reinitiate at GCN4 ORF downstream of the leader (reviewed in Gunišová et al., 2017). Likewise, during ribosome shunting in plant pararetroviruses, translation of the 5′-proximal uORF allows the post-terminating ribosomes to overcome the inhibitory effects of multiple downstream short uORFs and stable secondary structure and reinitiate translation of a long ORF downstream of the leader structure (discussed below).

Interestingly, short uORFs can regulate ribosomal sorting between two long ORFs, which results in translation of two proteins from the same mRNA (see below **Figure 3D**). Thus in the case of Kaposi’s sarcoma-associated herpesvirus, one of the viral mRNAs with overlapping long ORFs, ORF35, ORF36, and ORF37, is used for production of two proteins from ORF35 and ORF36 in a cap-dependent manner (Kronstad et al., 2013, 2014). Translation of ORF35 is achieved by leaky scanning through two short uORFs, uORF1, and uORF2, present in the 5′-leader sequence, because their AUG start codons are residing in weak and moderate initiation contexts, respectively. In contrast, translation of ORF36 is initiated by the ribosomes having translated uORF2: the 20 nt overlap between uORF2 and ORF35 is responsible for failure of the ribosomes terminating translation of uORF2 to initiate at the ORF35 start codon, and instead allows for downstream scanning and reinitiation at the ORF36 start codon (Kronstad et al., 2014). Notably, this mechanism ensures similar initiation frequencies at both ORF35 and ORF36, which is critical for viral lifecycle (Kronstad et al., 2013, 2014).

In simian immunodeficiency retrovirus (SIV), an uORF also regulates sorting of ribosomes between two long ORFs (van der Velden et al., 2012, 2013). SIV genomic RNA undergoes several splicing events producing monocistronic mRNAs for translation of each viral protein, except for the env protein, which is translated from a dicistronic mRNA containing the env ORF downstream of a rev ORF. In the 5′-leader of this mRNA, a highly conserved short uORF4 overlaps the downstream rev ORF. Owing to a moderate initiation context of the uORF4 start codon, leaky scanning through uORF4 allows for translation initiation at the rev ORF, while the ribosomes that do recognize and translate uORF4 can resume scanning further downstream and reinitiate at the env ORF. Additionally, the ribosomes may reach the env ORF by leaky scanning through both uORF4 and rev ORF initiation codons. Thus, the uORF4 is an important element of the leader that maintains balanced production of rev and env proteins (van der Velden et al., 2013). By analogy with SIV, uORF-mediated ribosomal sorting was proposed to operate in the human retrovirus HIV-1 for translation of vpu and env proteins from a dicistronic mRNA (van der Velden et al., 2012).

## Discovery of Ribosome Shunting

Fütterer et al. (1990a, 1993) discovered that a large middle portion of the 600-nt leader sequence of CaMV pgRNA containing several inhibitory uORFs and stable secondary structure is bypassed by scanning ribosomes. Such a non-linear ribosome migration combining features of 5′-end dependent scanning and internal initiation was named ribosome shunt or shunting (Fütterer et al., 1993). Subsequently, Fütterer et al. (1996) discovered that ribosome shunting also operates in RTBV, a CaMV-related plant pararetrovirus. The mechanism of CaMV shunt was further dissected by using plant protoplast and *in vitro* translation systems (Dominguez et al., 1998; Pooggin et al., 2000; Ryabova and Hohn, 2000; Ryabova et al., 2000) as well as *in planta* infectivity experiments with CaMV mutants (Pooggin et al., 1998, 2001). Likewise, the mechanism of RTBV shunt was eventually dissected and found to be very similar to that of CaMV (Pooggin et al., 2006). The ribosome shunt mechanisms in CaMV and RTBV, in comparison with other mechanisms of translation initiation, have been comprehensively reviewed in book chapters (Pooggin et al., 2002; Thiébeauld et al., 2007) and journal review articles (Ryabova et al., 2002, 2006). Here we review more recent findings, starting from 2008, which highlight the role of *cis*-elements driving ribosome shunting and the multipurpose use of pgRNA leader-based secondary structure and primary sequence elements in viral replication and infection cycles.

## Cross-Species Functionality of *cis*-Elements Driving Ribosome Shunting in Plant Pararetroviruses

**FIGURE 1:**
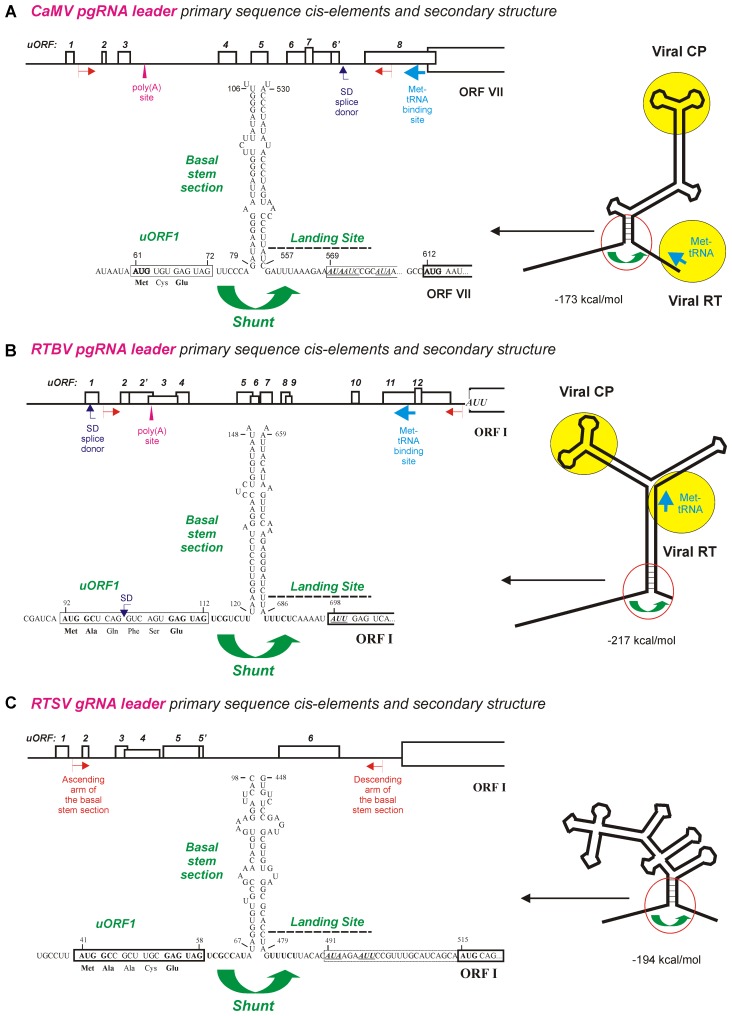
Ribosome shunt configurations in the pgRNA leaders of pararetroviruses CaMV and RTBV and the genomic (g)RNA leader of picorna-like virus RTSV. **(A–C)** Show the primary sequence *cis*-elements and secondary structures of CaMV, RTBV, and RTSV pgRNA leaders, respectively. The leader-based multiple uORFs preceding the main long ORF are numbered and positions of the poly(A) signal, the splice donor (SD), and the Met-tRNA primer binding site are indicated. The large stem-loop secondary structure formed by the leader sequence is shown schematically on the right with binding sites for the viral proteins CP (coat protein) and RT (reverse transcriptase) indicated by yellow circles. A close-up of the shunt configuration (red circle) is shown of the left, with primary sequences of the 5′-proximal uORF1 followed by the ascending arm of the basal stem-section and the descending arm of basal stem-section followed by the shunt landing sequence. The nt numbering starts from the leader 5′-end. For further details, please, see the main text. Adapted from Pooggin et al. (1999, 2012,2006).

Earlier bioinformatic analysis revealed a conserved shunt configuration in the pgRNA leader sequence of plant pararetroviruses (Pooggin et al., 1999). This configuration comprises the 5′-proximal short uORF terminating in front of the stable helical section of a large stem-loop secondary structure, which together represent the shunt take-off site, and an UA-rich unstructured sequence downstream of the stem-loop structure, which represents the shunt landing site. According to our model of shunt-mediated translation initiation on pararetroviral pgRNA (Ryabova et al., 2006; Thiébeauld et al., 2007), a 43S preinitiation complex binds the pgRNA capped 5′-end and scans along the leader sequence until the uORF start codon is encountered, where 60S joins and elongation begins. After translation of the uORF and a proper termination event at its stop codon, the released 40S shunts over the structured region and lands downstream of the structure, where it resumes scanning and re-initiates translation at a start codon of the first long viral ORF (**Figure 1**). It is assumed that the ATP-dependent RNA helicase complex (eIF4A/eIF4B) facilitating the scanning process by melting RNA secondary structures are lost during the first short translation event, which makes the released 40S subunit unable to melt the downstream structure and forces it shunt over the structured region and resume scanning at the unstructured landing sequence (Ryabova et al., 2006; Thiébeauld et al., 2007).

Both the bioinformatic analysis (Pooggin et al., 1999) and wet-lab investigations of shunt-mediated translation downstream of the CaMV and RTBV leaders in plant protoplasts and *in vitro* (Dominguez et al., 1998; Pooggin et al., 2000, 2001, 2006; Ryabova and Hohn, 2000; Ryabova et al., 2000) revealed that neither uORF-encoded peptide nor primary sequences composing the ascending and descending arms of the basal stem section are conserved or essential for ribosome shunting. Moreover, the uORF, the basal stem section arms and the UA-rich landing sequence could be swapped individually or in combination between CaMV and RTBV leaders without major effects on downstream translation (Pooggin et al., 2006, 2008). The most compelling evidence for cross-species functionality of the *cis*-elements driving ribosome shunt and for evolutionary conservation of the shunt configuration in dicot- and monocot-infecting pararetroviruses came from the infectivity studies of CaMV-RTBV chimera (Pooggin et al., 2008): a chimeric virus, in which two distant parts of the CaMV leader sequence that form the shunt configuration were replaced with the corresponding sequences from RTBV (**Figures 1A,B**), was infectious in turnip (CaMV-host) plants, albeit it exhibited a delay in systemic symptom development. Sequencing of the progeny viruses after several passages of the chimeric virus to new plants demonstrated an overall stability of the RTBV shunt configuration embedded in the CaMV leader, although single nt substitutions and short deletions did appear (Pooggin et al., 2008). Those alterations highlighted important features of the shunt take-off and landing sites, and showed fine tuning of the RTBV sequences evolving in the context of the distantly-related pararetrovirus CaMV. The most notable were short deletions at the chimeric junctions, upstream of the uORF and downstream of the landing site, which restored a relaxed secondary structure at both sites, likely facilitating the scanning ribosome to initiate uORF translation and the shunted ribosome to resume scanning and re-initiate translation. Furthermore, frequent mutations were found within the uORF coding sequence, which tended to inactivate a splice donor site (SD, see **Figure 1B**), without affecting the initiation and termination events at this uORF (Pooggin et al., 2008). In the context of RTBV infection, splicing of RTBV pgRNA fuses the leader-based uORF with a distal long ORF (ORF IV), thereby allowing translation of a viral protein P4 from the spliced RNA (Fütterer et al., 1994). Since a CaMV leader-based SD site is located at a different position (**Figure 1A**) and serves a different function (Kiss-László et al., 1995; discussed below), the additional SD site in the chimeric leader likely interfered with proper splicing of pgRNA and therefore was inactivated by point mutations in the viral progeny. Splicing described for *Caulimovirus* (CaMV) and *Tungrovirus* (RTBV) genera of *Caulimoviridae* and also expected to operate in some other genera (discussed below) enables or regulates expression of distal ORFs and at the same time regulates availability of unspliced pgRNA for translation and reverse transcription in the cytoplasm (Fütterer et al., 1994; Kiss-László et al., 1995; Froissart et al., 2004; Bouton et al., 2015), which illustrates complex interactions of plant pararetroviruses with the host nuclear machinery.

Integration of the RTBV shunt configuration into the CaMV leader region did not affect any other known *cis*-elements, namely, (i) the transcription and translation enhancers located between the transcription start site and the uORF start codon, (ii) the Met-tRNA primer binding site for initiation of reverse transcription located downstream of the shunt landing sequence, (iii) the poly(A) signal and (iv) the SD site located, respectively, at the ascending and descending arms of a middle section of the stem-loop structure, and (v) the pgRNA packaging signal exposed on an uppermost section of the stem-loop structure (**Figure 1A**; see Hohn and Rothnie, 2013 and references therein). Notably, a chimeric CaMV in which the uppermost stem-loop section with the packaging signal was replaced by the corresponding region from RTBV was not infectious in turnip plants, despite the replacement did not affect downstream translation in plant protoplasts (Pooggin et al., 2008). Thus, while the shunt configuration from RTBV could functionally substitute the CaMV shunt configuration in translation, replication, and systemic infection, the CaMV packaging signal that specifically binds the viral coat protein (CP) (Guerra-Peraza et al., 2000) could not be substituted with a putative pgRNA packaging signal from RTBV. This is likely because of structural differences in CPs of CaMV and RTBV that form icosahedral and bacilliform virions, respectively (Hohn and Rothnie, 2013).

## The Ribosome Shunt Configuration is Preserved in all Genera of Plant Pararetroviruses

**FIGURE 2:**
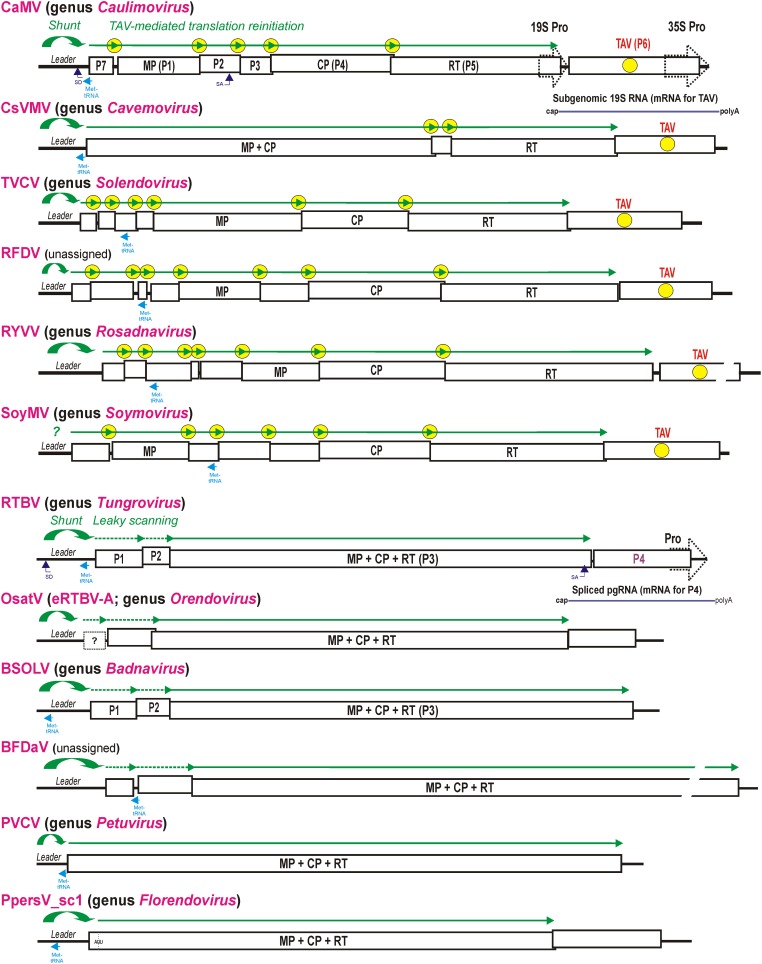
Polycistronic translation strategies in representative members of nine genera of the family *Caulimoviridae* and two unassigned caulimovirids. Organization of the viral pgRNAs is shown schematically with consecutive ORFs boxed and the conserved viral proteins (MP, CP, and RT) indicated. For CaMV and RTBV all the ORFs-encoded proteins are numbered (P1–P7 and P1–P4). In viral genera with TAV gene, the TAV protein-encoding ORF is indicated by yellow circle and the TAV-mediated translation reinitiation strategy is illustrated above pgRNAs by green lines with arrowheads indicating each translated ORF and yellow circles indicating reinitiation events. In the genera *Tungrovirus*, *Orendovirus*, *Badnavirus*, and in the unassigned BFDaV, which do not possess TAV, the RTBV-like leaky scanning strategy allowing translation of the MP-CP-RT ORF is illustrated with dotted green lines. The Met-tRNA binding site is highlighted in cyan. The splice donor (SD) and splice acceptor (SA) sites are indicated for CaMV and RTBV. The RTBV spliced RNA for P4 translation and the CaMV sgRNA for TAV/P6 translation are depicted. The two distinct groups of genera/viruses that differ by presence vs. absence of the TAV-encoding ORF and absence vs. presence of the MP-CP-RT polyprotein-encoding ORF, respectively, are grouped in upper and lower parts of the Figure, respectively. For further details, please, see the main text.

The family *Caulimoviridae* comprises eight recognized genera (*Badnavirus*, *Caulimovirus*, *Cavemovirus*, *Petuvirus*, *Rosadnavirus*, *Solendovirus*, *Soymovirus*, and *Tungrovirus*^1^), two unassigned members (*Blueberry fruit drop-associated virus*, BFDaV; Lockhart et al., 2017, and *Rudbeckia flower distortion virus*, RuFDV; Diaz-Lara and Martin, 2017), and two tentative genera of endogenous viral elements, *Orendovirus* (Geering et al., 2010) and *Florendovirus* (Geering et al., 2014) (**Figure 2**). The shunt configuration was so far identified in all examined members of the genera *Badnavirus*, *Caulimovirus*, *Cavemovirus*, *Petuvirus*, *Soymovirus*, and *Tungrovirus* (Pooggin et al., 1999; Geering et al., 2005; Rajeswaran et al., 2014b; Lim et al., 2015), with a notable exception for *Cestrum yellow leaf curling virus* from the genus *Soymovirus*, whose pgRNA leader sequence does fold into a stem-loop structure but lacks an uORF terminating in front of the stem (Stavolone et al., 2003). Other members of the genus *Soymovirus*, i.e., *Peanut chlorotic streak virus*, *Soybean chlorotic mottle virus* and *Blueberry red ringspot virus*, do possess a shunt configuration in the intergenic region, although the latter two viruses have a somewhat relaxed stem-loop structure and a 5′-proximal uORF with the minimal “start-stop” size (Pooggin et al., 1999). Thus, in the case of soymoviruses, leaky scanning and/or reinitiation after uORF translation can also contribute to translation initiation at the first long ORF downstream of the leader. Sequence inspection in members of the more recently recognized genera *Solendovirus* (*Sweet potato vein clearing virus* and *Tobacco vein clearing virus*) and *Rosadnavirus* (*Rose yellow vein virus*, RYVV) and the two unassigned caulimovirids (BFDaV and RuFDV) predicts shunt configurations in their intergenic region.

It is worth noting that a pgRNA leader-based stable stem-loop structure allows for prediction of an authentic start codon of the first long ORF, which is located downstream of the structure and can be either a standard AUG, as in the case of CaMV, or a non-AUG start codon, as in the case of RTBV and, likely, some members of the genus *Badnavirus* (Pooggin et al., 1999; Geering et al., 2005; Rajeswaran et al., 2014b).

The tentative genus *Florendovirus* contains multiple viral species integrated into the genomes of many flowering plants (Geering et al., 2014) and, by species diversity, it is comparable to the genus *Badnavirus.* The latter genus also contains endogenous viral elements, some of which can be released from the host genome as infectious and transmissible episomal viruses (reviewed in Chabannes and Iskra-Caruana, 2013). In *Prunus persica*, one of the *Florendovirus* loci, namely PpersV-sc1, contains a potentially infective partial dimer with an uninterrupted copy of the virus genome with complete conserved ORFs (Geering et al., 2014) (**Figure 2**). A predicted pgRNA leader sequence of PpersV-sc1 folds into a stable stem-loop structure with a 5′-proximal short uORF terminating in front of the stem, suggesting that translation of the first large ORF encoding a MP-CP-RT (movement protein-coat protein-reverse transcriptase) polyprotein (ORF I) downstream of the leader is initiated by ribosome shunting. It would be interesting to explore if PpersV-sc1 and perhaps other florendoviruses with uninterrupted genome copies and conserved shunt configurations are still infective.

The tentative genus *Orendovirus* contains three putative RTBV-like species which had been integrated into the rice genome long time ago and then decayed through mutation and recombination into multiple fragments that cannot give rise to an infective episomal virus (Kunii et al., 2004; Geering et al., 2010). Nonetheless, reconstruction of their potential ancestral genomes from the fragments reveals three ORFs resembling the ORFs I (P1), III (MP-CP-RT), and IV (P4) of RTBV (Kunii et al., 2004) and a large intergenic region containing a shunt configuration (**Figure 2**). Taken together, a ribosomal shunt configuration appears to have evolved in a progenitor pararetrovirus, possibly from an inverted repeat in the intergenic region, and ever since been preserved in all genera of the *Caulimoviridae*.

## Co-Evolution of the Ribosome Shunt Elements in RTBV and an RNA Virus RTSV Forming a Stable Tungro Disease Complex

The pararetrovirus RTBV and the RNA picorna-like virus *Rice tungro spherical virus* (RTSV) (genus *Waikavirus*, family *Secoviridae*) form a stable complex associated with rice tungro disease (reviewed in Hull, 1996). In this unique complex, duties are divided between the partners: RTBV is responsible for disease symptom development, possibly associated with RTBV P4-mediated suppression of plant RNAi (Rajeswaran et al., 2014a; discussed below), while RTSV is responsible for leafhopper-mediated transmission of both viruses from plant to plant (Hull, 1996). Such intimate relationships suggest that during co-existence within a stable disease complex the two viruses might have co-evolved regulatory *cis*-elements to coordinate their replication in rice cells and systemic movement through vascular system where they are acquired by sap-sucking leafhoppers for transmission. This hypothesis has begun to be supported since an RTBV-like ribosome shunt was found to operate during translation of RTSV genomic RNA, which requires proper translation initiation and termination at the 5′-proximal short uORF and formation of a stable basal section of the large stem-loop structure just downstream of the uORF stop codon (Pooggin et al., 2012; **Figure 1C**).

Like RTBV, RTSV has a long 5′-leader sequence with several short uORFs that can fold into the stable stem-loop structure (**Figures 1B,C**): these features are known to contribute to inhibition of downstream translation as was demonstrated for CaMV (Pooggin et al., 2000). In both RTBV and RTSV, the 5′-proximal uORF is short (7 and 6 codons), has a moderate start codon context (UCA AUG GCU and CUA AUG GCA), terminates at a short distance (7 and 8 nts) in front of the stem-loop basal helix and has an identical stop codon context (GAG UAG UCG). Moreover, the shunt landing sequences downstream of the basal stem-section are strikingly similar: both are UA-rich, have a low index of secondary structure and a non-AUG start codon at similar distance from the stem (**Figures 1B,C**). In RTBV, the non-AUG start codon AUU in the shunt landing site is recognized by about 10% of shunted ribosomes to initiate translation of the first long viral ORF (ORF I) as shown in rice protoplasts (Fütterer et al., 1996). Likewise, two non-AUGs in the CaMV landing sequence are recognized by shunted ribosomes, albeit with low efficiency as demonstrated *in vitro* (Ryabova and Hohn, 2000). It was proposed that after uORF translation the shunting ribosomes loose fidelity factors responsible for proper recognition of an AUG start codon. Interestingly, the RTSV landing sequence contains two non-AUG codons and both are in frame with a downstream AUG start codon of the large ORF encoding a polyprotein (**Figure 1C**), suggesting that a fraction of the viral polyprotein may have an N-terminal extension(s). Another common feature of the RTBV and RTSV shunt landing sites is a pyrimidine tract with the identical sequence UUUCU located a few nts upstream of the non-AUG start codon (**Figures 1B,C**): this tract can potentially facilitate non-AUG recognition by analogy with internal ribosome entry sites of animal picornaviruses (Belsham, 2009). It should be noted that, in the picorna-like virus RTSV, internal initiation of translation was ruled out in favor of ribosome shunting, because translation downstream of the RTSV leader was strictly dependent of a proper translational event at the 5′-proximal uORF and was abolished by insertion at the 5′-end of the leader sequence of a strong and compact structure (Kozak-stem) that blocks 5′-end-dependent scanning (Pooggin et al., 2012).

The striking similarity and even identity in the primary sequence elements at the shunt take-off and landing sites of RTBV and RTSV (**Figures 1B,C**) imply that those elements in RTSV have evolved after encounter of two viruses in one host plant, presumably through recombination and further co-evolution into a stable complex. In favor of this hypothesis, other members of the family *Secoviridae* do not appear to have evolved an RTSV-type shunt configuration, although the waikavirus *Maize chlorotic dwarf virus* does possess a long leader with several uORFs (Pooggin et al., 2012).

It is puzzling why RTSV would need to evolve a sophisticated ribosome shunt mechanism by enlarging its genomic RNA leader sequence and making it a major obstacle for scanning ribosomes (Pooggin et al., 2012). In RTBV (by analogy with CaMV), the highly-structured leader region, shunted over during translation initiation, contains the putative RNA packaging signal, a purine-rich sequence exposed on top of the large stem-loop structure for interaction with viral CP (Guerra-Peraza et al., 2000; **Figures 1A,B**): the viral CP binding to the packaging signal, preserved by shunting ribosomes from being melted, would allow to divert the pgRNA from translation to packaging into a previrion, followed by reverse transcription (Pooggin et al., 1999; Ryabova et al., 2002; Schoelz and Leisner, 2017; references therein). It would be interesting to explore if RTSV had co-evolved a similar mechanism for sorting its genomic RNA for translation and packaging and thereby coordinating its replication cycle with RTBV in co-infected rice cells. Additionally, RNA secondary structure can potentially be protective against repressive action of virus-derived siRNAs generated by the plant RNAi machinery (discussed below).

## Ribosome Shunting in Animal Viruses

Another example of an uORF-containing shunt configuration was discovered in a leader region of *Prototype foamy virus* (PFV) (Schepetilnikov et al., 2009). PFV has an obscure origin, being isolated from a human patient and originally named *Human foamy virus* (HFV) but then found to be very similar to *Simian foamy virus* (SFV) isolated from chimpanzees. The lab strain of PFV can infect all vertebrate cells tested, from human to fish. Foamy viruses belong to the subfamily *Spumaretrovirinae* and differ from other retroviruses of the family *Retroviridae*, because their replication strategy combines features of both true retroviruses (subfamily *Orthoretrovirinae*) and pararetroviruses from the families *Caulimoviridae* and *Hepadnaviridae* (animal pararetroviruses) (Lindemann and Rethwilm, 2011; Rethwilm and Bodem, 2013). Besides reverse transcription of pgRNA, PFV and CaMV share other common features. Like CaMV, PFV has two promoters, where the second internal promoter drives transcription of accessory transactivation functions (Lindemann and Rethwilm, 2011). Similar to CaMV, PFV employs a ribosome shunt mechanism to initiate translation of Gag/CP ORF downstream of the leader (Schepetilnikov et al., 2009). The 445 nt leader sequence of PFV pgRNA forms a stable stem-loop structure and contains two phylogenetically-conserved uORFs, one (uORF A/A′) terminating in front of the basal stem section and another (uORF B) in the ascending arm of the stem-loop structure. Interestingly, the efficiency of shunting depends on stability of the stem section located downstream of either uORFs A/A′ or uORF B, respectively, and on the translation event at the corresponding uORF (Schepetilnikov et al., 2009). This implies two shunting events, one after translation of uORF A/A′ and another after translation of uORF B, which would ensure preservation of the stable secondary structure with *cis*-elements predicted to be involved in pgRNA packaging (Schepetilnikov et al., 2009). Likewise, a second, less-efficient, shunting event was shown to operate in the CaMV leader which depends on the second uORF terminating in front of the stem-section 2 (Ryabova et al., 2000).

Other examples of ribosome shunting previously described for mammalian viruses and cellular mRNAs indicate that the mammalian ribosomes can bypass 5′-leader sequences that do not possess a CaMV-like shunt configuration (reviewed in Ryabova et al., 2002, 2006). Thus, an uORF-independent ribosomal shunt operating on a tripartite leader of adenovirus late mRNAs depends on *cis*-elements with complementarity to the 3′-end of 18S rRNA and several hairpin-like secondary structures, which appear to slow down the scanning ribosome and provide conformation essential for shunting (Yueh and Schneider, 1996, 2000). Although both linear scanning and shunting operate on the tripartite leader during the early phase of infection, shunting becomes the only mechanism of initiation during the late stage of infection (Yueh and Schneider, 1996) and a 100 kD adenoviral protein promotes the shunting process by specifically binding eIF4G (Xi et al., 2005).

In the duck hepatitis B pararetrovirus (family *Hepadnaviridae*), pgRNA is used as a bicistronic mRNA with two consecutive long ORFs encoding the core/CP and Pol/RT (RT) proteins. Translation of the Pol protein is initiated by ribosome shunting from an undefined take-off site near the 5′ end of pgRNA comprising the stem-loop with a packaging/reverse transcription signal 𝜀 to at least two landing sites near the Pol ORF AUG start codon. In this case, secondary structure(s) and possibly two short uORFs (C01/C02) upstream of the core ORF contribute to shunt efficiency (Sen et al., 2004; Cao and Tavis, 2011). Interestingly in the human hepatitis B pararetrovirus, a highly-conserved short uORF (C0), whose AUG start codon resides in a moderate initiation context, overlaps the core ORF and appears to regulate optimal production of the core and Pol proteins (Chen et al., 2005). It would be interesting to investigate if ribosome shunting operates in this case and in other animal pararetro- and retroviruses possessing regulatory uORFs and/or secondary structure elements.

## Polycistronic Translation in Plant Pararetroviruses

With a notable exception for the genus *Petuvirus* with its single member *Petunia vein clearing virus* encoding one large polyprotein ORF, all the other genera of *Caulimoviridae* have pgRNAs with two or more long consecutive ORFs connected to each other by short overlaps, sometimes with fused stop and start codons, or short intervening sequences (**Figure 2**; discussed below). Such genome organization implies polycistronic translation. Based on available experimental evidence, at least two strategies of polycistronic translation have been evolved in the family *Caulimoviridae*: (i) leaky scanning through long ORFs devoid of internal AUGs and (ii) viral protein-activated translation reinitiation after long ORFs.

**FIGURE 3:**
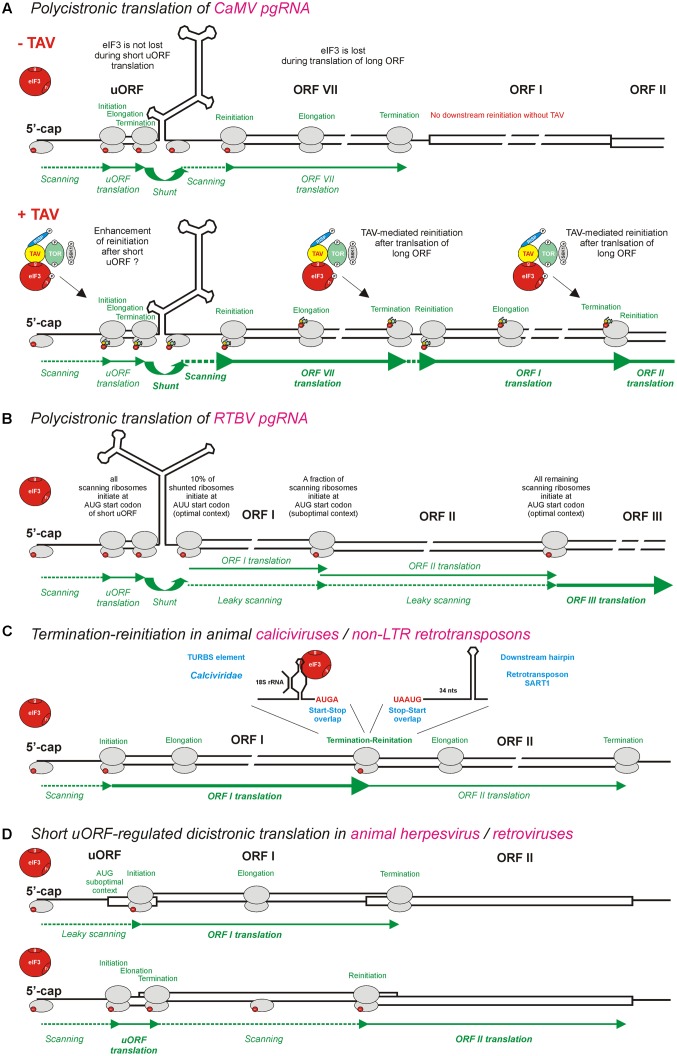
Models for polycistronic translation in plant pararetroviruses CaMV and RTBV and in animal viruses and non-LTR retrotransposons. **(A,B)** Illustrate schematically the viral protein TAV-mediated reinitiation and the leaky scanning mechanisms operating on pgRNAs of CaMV and RTBV, respectively. **(C)** Illustrates the termination-reinitiation mechanisms operating in animal caliciviruses and non-LTR retrotransposons. **(D)** Illustrates the ribosomal sorting mechanisms operating in animal herpesvirus and pararetroviruses. eIF3 (red circle) is shown as the key initiation factor involved in polycistronic translation. Note that eIF3 alone or together with other factors is also shown in smaller scale on 40S or 60S ribosomal subunits (gray ovals). The CaMV TAV, through its interactions with eIF3, TOR, and RISP, keeps eIF3 attached to the translating ribosome during long ORF translation (see **Figure 4**) and thus promotes several consecutive reinitiations by post-terminating ribosomes. For further details, please, see the main text.

Leaky scanning was discovered in RTBV by Fütterer et al. (1997), who demonstrated that, about 10% of the ribosomes that bypass the pgRNA leader structure by shunting, initiate translation at the AUU start codon of ORF I (Fütterer et al., 1994), while the remaining 90% scan through the AUU and reach the AUG start codon of ORF II. A moderate context of the ORF II AUG also allows for a substantial fraction of the ribosomes to scan through this codon and reach the AUG of ORF III (**Figure 3B**). The leaky scanning process for a distance of 900 nts on the RTBV pgRNA is facilitated by the absence of internal AUG start codons within the ORFs I and II (Fütterer et al., 1997).

By analogy with RTBV, leaky scanning can also be predicted to operate in members of the genus *Badnavirus*, which resemble RTBV in the organization of ORF I (P1), II (P2), and III (MP-CP-RT) (**Figure 2**) and also keep the coding sequences of ORFs I and II free of internal AUGs (Fütterer et al., 1997; Pooggin et al., 1999; Rajeswaran et al., 2014b). Likewise, the unassigned caulimovirid BFDaV has genome organization similar to members of the genus *Badnavirus*, and its pgRNA is presumably translated via ribosome shunting and leaky scanning through weak AUG start codons of two long ORFs preceding the MP-CP-RT polyprotein-encoding ORF (**Figure 2**). Notably, in BFDaV and some members of the genus *Badnavirus*, one or two short uORFs are present within ORF I, but their AUG codons are in a suboptimal context, similar to the start codons of ORFs I and II, and may therefore allow for leaky scanning. In RTBV, mutation of the ORF I AUU start codon to AUG or strengthening the context of the ORF II AUG both drastically reduced ORF III translation, whereas introduction of a short uORF within ORF II did not affect ORF III translation (Fütterer et al., 1997).

Unlike other members of the genus *Badnavirus*, *Sweet potato pakakuy virus* (SPPV) was reported to have a split ORF III (Kreuze et al., 2009; Genbank accession NC_015655.1). Inspection of its predicted pgRNA reveals a long leader with several uORFs and a stem-loop structure, followed by the coding sequences of ORFs I and II devoid of internal AUGs, the features compatible with ribosome shunting and leaky scanning. However, the region between the ORF IIIa and the ORF IIIb AUG start codons contains multiple internal AUGs including those with optimal initiation contexts, making leaky scanning through this region unlikely. Since SPPV and other badnaviruses do not encode any CaMV-like transactivator of translation after long ORFs (discussed below), a strategy of ORF IIIb translation remains unclear. Because no infectious clone of SPPV is available, it cannot be formally excluded that the interrupted ORF III is an artifact of sequencing. Indeed, this virus was detected and assembled by small RNA sequencing, followed by PCR to bridge the small RNA contigs (Kreuze et al., 2009), which cannot rule out PCR amplification of sweet potato genome-integrated SPPV-like sequences with interrupted ORF III.

Transactivation of polycistronic translation on pgRNA by a viral TAV protein was co-discovered by Bonneville et al. (1989) and Gowda et al. (1989) in CaMV and *Figwort mosaic virus* (FMV), the members of genus *Caulimovirus*, and then confirmed for *Peanut chlorotic stunt virus* from genus *Soymovirus* (Maiti et al., 1998). The studies of CaMV and FMV gene expression in plant protoplasts have demonstrated that *in trans* expression of TAV is required for translation of ORFs located downstream of ORF VII, which is adjacent to the pgRNA leader sequence and itself translated via a ribosomal shunt mechanism. It should be noted that shunt-mediated translation initiation at ORF VII does not require TAV, albeit it can be enhanced by TAV (Fütterer et al., 1993). In contrast, TAV is absolutely essential for translation of ORF I located downstream of ORF VII (Bonneville et al., 1989; Gowda et al., 1989) as well as further downstream ORFs II, III, IV, and V (Fütterer et al., 1990b; Scholthof et al., 1992). ORFs I and II are likely translated from pgRNA via sequential TAV-mediated reinitiation, following termination of translation at the stop codons of ORFs VII and I, respectively (**Figure 3A**; discussed below). This mechanism can potentially allow further downstream translation of ORFs III (P3), IV (P4/CP) and V (P5/RT) on pgRNA (**Figure 2**), although those three ORFs are more likely translated from the spliced pgRNA, in which the spicing event using a SD site in the CaMV leader sequence and a SA site within ORF II (P2) makes ORF III the first long ORF downstream of a shortened leader sequence (Kiss-László et al., 1995) (see the SD and SA sites in **Figure 2**).

As shown for CaMV, the TAV/P6 protein itself is translated from a monocistronic subgenomic RNA (sgRNA), 19S RNA, transcribed by Pol II from a separate 19S promoter sharing a transcriptional enhancer with the 35S promoter that drives Pol II-mediated transcription of the 35S pgRNA (Driesen et al., 1993) (**Figure 2**, top). This strategy is likely exploited in all members of the genus *Caulimovirus* as well as other genera of plant pararetroviruses encoding a TAV homolog at a similar genome position. The *Caulimovirus* TAV homologs are encoded in members of the genera *Cavemovirus*, *Solendovirus*, and *Soymovirus* as well as in the unassigned caulimovirid RFDV (**Figure 2**), suggesting that in those pararetroviruses TAV-mediated transactivation would allow polycistronic translation of pgRNA and its potentially spliced versions. In contrast, no homolog of TAV is recognizable by protein BLAST in the genera *Petuvirus, Badnavirus, Tungrovirus, Orendovirus, Rosadnavirus*, or *Florendovirus*. In the tentative genus *Florendovirus*, pgRNA containing two large ORFs (**Figure 2**) potentially serves as a monocistronic mRNA for an MP-CP-RT polyprotein encoded by ORF I, while a downstream ORF II is presumably translated either from a sgRNA transcribed from its own promoter (like TAV-encoding ORFs in *Caulimovirus, Soymovirus*, *Cavemovirus, Rosadnavirus*, and *Solendovirus*), or from a spliced pgRNA (like in the case of tungrovirus RTBV and possibly RTBV-like orendoviruses). In the *Rosadnavirus* RYVV, the genome organization implies that reinitiation after long ORFs would be required to translate its polycistronic pgRNA (**Figure 2**).

## The Mechanism of TAV-Mediated Reinitiation after Translation of Long ORFs

The reason why eukaryotic ribosomes cannot normally translate two or more consecutive long ORFs on one mRNA is likely related to availability of reinitiation-promoting factors that are required for *de novo* acquisition, by the 40S ribosomal subunit released at the stop codon, of the ternary complex and 60S for the next initiation event. ORF length-dependent decrease in downstream reinitiation suggests only temporary retention of eIFs on the translating ribosome due to their gradual loss during the elongation phase (Fütterer and Hohn, 1992; Kozak, 2001; Pöyry et al., 2004). The critical translation initiation factor eIF3 that promotes nearly all initiation steps including scanning and AUG recognition (Hinnebusch, 2006; Jackson et al., 2010; Browning and Bailey-Serres, 2015) was suggested as the key reinitiation-promoting factor (Park et al., 2001; Pöyry et al., 2004; Hinnebusch, 2006). Indeed, recently it was documented *in vivo* that eIF3 remains attached to the ribosome during translation of short uORFs 1 and 2 on the yeast GCN4 mRNA, while it dissociates from the ribosome translating elongated uORFs (Mohammad et al., 2017). Temporary retention of eIF3 and perhaps other factors might help the ribosome having translated a short uORF to rapidly reacquire the ternary complex to become reinitiation-competent. In addition to eIF3, the initiation factor eIF4F, the elongation factor eEF2 and the non-canonical initiation factor DENR-MCT-1 have been implicated in reinitiation after short uORF translation in yeast and mammals (Cuchalová et al., 2010; Munzarová et al., 2011; Skabkin et al., 2013; Schleich et al., 2014; reviewed in Gunišová et al., 2017). In plants, the eIF3 subunits g (Park et al., 2001) and h (Kim et al., 2004; Schepetilnikov et al., 2013) and the 60S ribosomal protein eL24 (Nishimura et al., 2005) appear to be required to overcome the inhibitory effects of short uORFs.

The protein–protein interaction analysis revealed that the CaMV TAV/P6 associates with eIF3 via its subunit g and with the 40S ribosomal subunit via eIF3 as a bridge (Park et al., 2001). However, eIF4B can preclude the formation of 40S/eIF3/TAV complex by competition with TAV for eIF3g binding, suggesting that TAV enters the translation machinery after eIF4B removal from 40S (Park et al., 2004). In addition, TAV can interact with the 60S ribosomal subunit via at least three ribosomal proteins eL24, eL18, and eL13 (Leh et al., 2000; Park et al., 2001; Bureau et al., 2004; Thiébeauld et al., 2009). Such interaction network suggests that TAV can stabilize the complex between eIF3 and the ribosome during repeated reinitiation events (**Figure 3A**; discussed below). This hypothesis is supported by the fact that actively translating ribosomes (polysomes) are highly enriched in eIF3 and TAV in the Arabidopsis plants transgenic for TAV (Schepetilnikov et al., 2011).

Another co-factor that promotes TAV-mediated reinitiation after long ORF translation is a reinitiation supporting protein (RISP). RISP interacts not only with TAV, but also with the TAV partners eIF3 (via its subunits a and c) and eL24 (via its C-terminus) in the presence or absence of TAV (Thiébeauld et al., 2009). In wheat germ, endogenous RISP associates with salt-washed 80S monoribosomes and 60S ribosomal subunits (Thiébeauld et al., 2009). Notably, RISP binds the C-terminus of eL24, while TAV interacts with the N-terminus of eL24, suggesting the existence of a complex between TAV-RISP and 60S. Indeed, both TAV and RISP are found in the polysomes from CaMV-infected plants (Schepetilnikov et al., 2011), and both eIF3 and RISP accumulate to high levels in the polysomes from Arabidopsis transgenic seedlings expressing the wild type TAV (Thiébeauld et al., 2009), suggesting that, in the presence of TAV, eIF3 and RISP are bound to the translating ribosomes.

## TAV Binds and Activates TOR Kinase Required for Reinitiation after Long ORF Translation

Under conditions of nutrient and energy sufficiency the activity of the protein kinase target of rapamycin (TOR) promotes cell growth and blocks autophagy, while TOR inactivation under starvation conditions induces autophagy to increase levels of nutrient pools and energy (Robaglia et al., 2012; González and Hall, 2017; Pu et al., 2017). TOR is a key controller of cap-dependent translation initiation in mammals (Pelletier et al., 2015). In plants, TOR was implicated in translation of mRNAs harboring uORFs in their leader regions (uORF-mRNAs), and found to attenuate the inhibitory effects of these short uORFs (Schepetilnikov et al., 2013). Auxin was identified as an upstream effector of TOR, which activates TOR via a small GTPase ROP2 (Schepetilnikov et al., 2017). The auxin-ROP2-TOR signaling facilitates translation of uORF-mRNAs, in part, via phosphorylation of the subunit h of eIF3 (Schepetilnikov et al., 2017; reviewed in Schepetilnikov and Ryabova, 2017a). Interestingly, mammalian TOR (mTOR) binds the eIF3-40S complex and can use it as a platform for phosphorylation of the ribosomal protein S6 kinase 1 (S6K1) (Holz et al., 2005). According to the authors’ model, inactive S6K1 associates with eIF3 within the 48S preinitiation complex, while inactive mTOR does not. Upon activation, mTOR is recruited to eIF3, where it phosphorylates S6K1, triggering its dissociation from eIF3 followed by further phosphorylation by PDK1 (Holz et al., 2005). In plants, the polysomes, in addition to eIF3-containing preinitiation complexes, serve as a platform for TOR phosphorylation events (Schepetilnikov et al., 2013). Auxin signaling promotes active TOR binding to the inactive S6K1-prebound eIF3-containing complexes and the polysomes, which leads to phosphorylation of S6K1 at its hydrophobic motif residue Thr449 (Schepetilnikov et al., 2013). The phosphorylated S6K1, released from both platforms, becomes fully activated and capable to phosphorylate eIF3h. Notably, the patterns of association with TOR are opposite to those of S6K1.

In addition to facilitating reinitiation after short uORF translation, TOR assists TAV in transactivation of reinitiation after translation of long ORFs. Comparative analysis of transgenic plants expressing the wild type CaMV TAV and its inactive versions revealed that TAV can physically interact and activate TOR (Schepetilnikov et al., 2011). Notably, the TOR-binding domain of TAV (designated “dsR,” discussed below) is located within “mini-TAV,” a minimal portion of the CaMV P6/TAV protein that retains a residual transactivaton activity (de Tapia et al., 1993). TAV-mediated hyperactivation of TOR leads to phosphorylation and activation of its downstream target S6K1. The reinitiation supporting protein RISP (Thiébeauld et al., 2009) was identified as a novel substrate of S6K1 that is phosphorylated at S267 in a TOR-responsive manner (Schepetilnikov et al., 2011). Both TOR and phosphorylated RISP associate with polysomes in TAV-transgenic plants. In contrast, the TAV mutant defective in TOR binding and thus in TAV-mediated reinitiation fail to recruit TOR to polysomes, which in this case can still associate with non-phosphorylated RISP. Accordingly, phosphorylated RISP preferentially binds TAV and stimulates TAV-mediated reinitiation (Schepetilnikov et al., 2011). These findings suggest that a functional role of TOR in TAV-mediated reinitiation would be to maintain the high phosphorylation status of RISP and possibly other reinitiation-supporting factors on the polysomes. Consistent with the key role of TOR, mesophyll protoplasts prepared from TOR-deficient Arabidopsis plants failed to promote reinitiation after translation of long ORFs with or without TAV, and, as a consequence, TOR-deficient plants were found to be resistant to CaMV whose life cycle depends on TAV-mediated polycistronic translation (Schepetilnikov et al., 2011).

**FIGURE 4:**
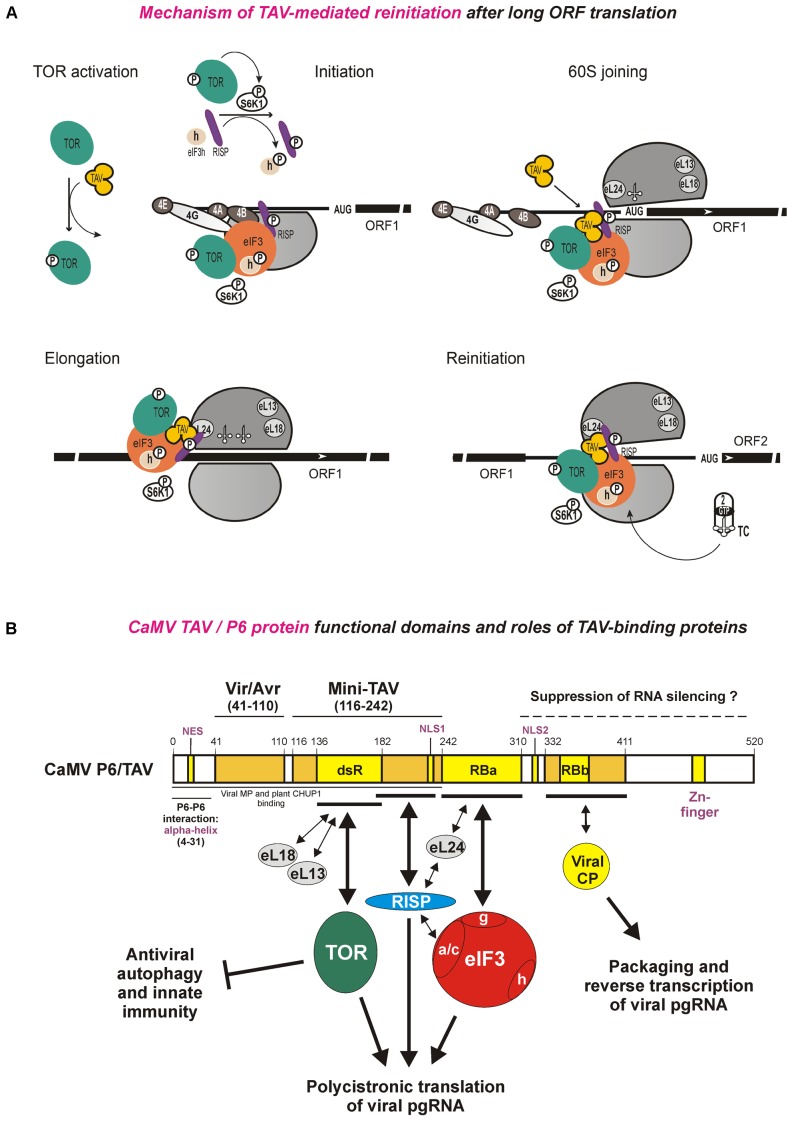
Model for CaMV TAV function in reinitiation after long ORF translation and the functional domain structure of TAV protein with its interacting protein factors. **(A)** The mechanism of TAV-mediated translation reinitiation divided in five phases. At the TOR activation phase, TAV binds and activates TOR. At Initiation phase, activated TOR binds eIF3-40S preinitiation complex at the mRNA 5′-cap to trigger S6K1 and thus RISP and eIF3h phosphorylation. At 60S joining phase, eIF4B is replaced by TAV on eIF3g, and TAV forms a complex with TOR-eIF3-RISP on 80S. During Elongation phase, TAV allows TOR-eIF3-RISP retention on the translating ribosome, through interaction with eL24 at the periphery of the 60S interface. At Reinitiation phase, following termination of long uORF translation, the putative TOR-eIF3-TAV-RISP complex is relocated back to 40S via eIF3-40S interactions. TAV and RISP bridge the 40S-bound eIF3 and the 60S-bound eL24, respectively, such that the post-terminating 60S remains attached to 40S and can be reused for the reinitiation event. The reinitiation-competent 40S-containing complex carrying 60S is capable of resuming scanning, rebinding a ternary complex (TC) and reinitiating at downstream ORF. eIF4E (4E), eIF4G (4G), eIF4A (4A), eIF4B (4B), eIF2 (2), tRNA and the ternary complex (TC, eIF2/GTP/Met-tRNAi^Met^) are indicated. Joining of TAV, recycling of TC and phosphorylation of S6K1 and RISP or eIF3h is shown by arrows. For further details, please, see the main text. Adapted from Thiébeauld et al. (2009),Schepetilnikov et al. (2011). **(B)** CaMV TAV interaction network illustrating functional domains of the TAV/P6 protein and roles of the TAV-binding cellular (TOR, RISP, eIF3, eL13, eL18, and eL24) and viral (CP and MP) proteins in polycistronic translation and packaging/reverse transcription of CaMV pgRNA and in suppression of antiviral defenses. For further details, please, see the main text.

Our current model for TAV-mediated reinitiation after long ORF translation is shown in **Figure 4A**, which illustrates the roles of all the TAV-binding and reinitiation-supporting factors described above. The model presents five phases: TOR activation, Initiation, 60S joining, Elongation and Reinitiation (**Figure 4A**) and is assisted by the TAV interaction network shown in **Figure 4B**. Based on the observation that RISP co-immunoprecipitates with eIF3 and eIF2 *in planta* and, together with eIF3, binds 40S (Thiébeauld et al., 2009), RISP likely enters the translation machinery together with eIF3 at the stage of 43S preinitiation complex formation at the capped 5′end of mRNA (**Figure 4A**, Initiation phase). TOR, activated by TAV, is then loaded on the 43S preinitiation complex, where it can phosphorylate S6K1 and thus mediate phosphorylation of RISP and the subunit h of eIF3 (**Figure 4A**, Initiation phase). TAV itself joins the initiating ribosome at the 60S joining step via binding to the 40S-associated eIF3 following, or concomitant with, disruption of eIF3g interaction with eIF4B, and, through its interactions with eIF3g, phosphorylated RISP and TOR, TAV stabilizes an unstable TOR-eIF3-RISP complex which thus remains attached to the ribosome (**Figure 4A**, 60S joining phase). Indeed, the binding domains for TOR, RISP, and eIF3g are not overlapping on the TAV/P6 protein (**Figure 4B**). Based on the observation that TOR, when activated by TAV or auxin, is loaded onto polysomes, active TOR may also be loaded on the polysomes directly, where it can contact the eIF3-TAV-RISP complex likely via eIF3 or TAV and maintain S6K1 phosphorylation and thus the high phosphorylation status of RISP and eIF3h. Following the 60S joining step, a putative TOR-eIF3-TAV-RISP complex can stay associated with the translating ribosomes for a long time during elongation, likely by attaching to the 60S subunit (**Figure 4A**, elongation phase). Taking in account that both eL18 and eL13 are located in relatively close proximity on the external surface of 60S below the neck region, while eL24 is located at the periphery of 60S with its C-terminal alpha-helix protruding out of 60S (Klinge et al., 2011), TAV-containing complex(es) can be relocated from 40S to 60S via binding to either eL24 or eL18/eL13. Despite that all these three ribosomal proteins are easily accessible for external interactions on the 80S ribosome (Ben-Shem et al., 2011; Klinge et al., 2012), we assume that the putative TOR-eIF3-TAV-RISP complex is more likely connected to 60S via eL24 (**Figure 4A**, elongation phase). Both RISP and TAV can form a stable complex with eL24 and thus retain eIF3 and TOR on 60S via eL24-RISP-eIF3 and eL24-TAV-TOR links (for interaction details see **Figure 4B**). In contrast, interaction of TAV complex(es) with eL18/eL13 might be precluded by TOR, since the same dsR domain within mini-TAV is involved in binding eL18 (Leh et al., 2000), eL13 (Bureau et al., 2004), and TOR (Schepetilnikov et al., 2011) (**Figure 4B**). Upon termination of translation, eIF3 is relocated back to 40S to allow reinitiation of translation (Park et al., 2001, 2004). At the reinitiation phase, according to a “60S re-cycle” hypothesis proposed based on the RISP interaction network (Thiébeauld et al., 2009), the function of the eIF3-TAV-RISP complex would be to bridge the relaxed 40S–60S interactions through dynamic contacts with both 40S via eIF3 and 60S via eL24 during the resumption of scanning toward the downstream initiation codon (**Figure 4A**, reinitiation phase). Although binding of eIFs 3, 1 and 1A to the post-termination complex normally causes 60S to dissociate from the mRNA *in vitro* (Pisarev et al., 2007), dissociation of 60S during the termination event would be delayed in the presence of TAV, thus promoting 60S re-joining during the reinitiation event. The 60S re-cycle hypothesis (Thiébeauld et al., 2009) explains why the efficiency of TAV-activated polycistronic translation is not dependent on the distance between two ORFs (Fütterer and Hohn, 1991, 1992), as TAV-RISP would promote 60S re-use by keeping 60S associated with 40S during the scanning to a downstream ORF (**Figure 4A**; reinitiation phase).

Taken together, TAV plays a dual function in reinitiation after translation of long ORFs: it maintains association of eIF3 and RISP with the translating ribosomes during multiple long elongation cycles and activates TOR kinase to ensure the functional active state of RISP and perhaps other reinitiation factors. As mentioned above, TOR activation in response to auxin leads to phosphorylation of the subunit h of eIF3, thereby facilitating translation reinitiation on mRNAs containing leader-based short uORFs (Schepetilnikov et al., 2013; reviewed in Schepetilnikov and Ryabova, 2017b). It is conceivable that TAV-mediated activation of TOR would also lead to phosphorylation of eIF3h and thereby facilitate reinitiation at the first long viral ORF by the shunting ribosomes, following translation of the 5′-proximal short uORF on the CaMV pgRNA leader (**Figure 3A**). This hypothesis is consistent with the finding that CaMV TAV enhances the efficiency of ribosome shunting about two–threefold (Fütterer et al., 1993; Pooggin et al., 2008). Notably, TAV-mediated enhancement of ribosome shunting boosts further downstream consecutive reinitiation events on the polycistronic pgRNA (**Figure 3A**).

## Termination-Reinitiation in Animal Viruses and non-LTR Retrotransposons

So far no evidence is available for existence of any TAV-like reinitiation activator protein or virus-activated transactivation of polycistronic translation outside of the family *Caulimoviridae*. Interestingly, animal caliciviruses and noroviruses of the family *Caliciviridae* have evolved a termination-reinitiation mechanism, which allows translation of two consecutive ORFs connected by a start-stop codon overlap (AUGA) and depends on a termination upstream ribosome binding site (TURBS) element of 40–87 nts located shortly upstream of the stop-start overlap (Meyers, 2007; Luttermann and Meyers, 2009; Luttermann and Meyers, 2014). The TURBS element was suggested to function by promoting direct interactions between mRNA and 18S rRNA (the component of 40S ribosomal subunit) that would delay the 40S dissociation after termination of translation. Notably, the termination-reinitiation mechanism appears to be further facilitated by the interaction of TURBS with eIF3 (Pöyry et al., 2007). Thus the interactions of TURBS with both 40S (through 18S rRNA) and eIF3 would allow the post-terminating ribosome to re-acquire eIF3 and the ternary complex (both being lost during long uORF translation) and reinitiate translation (**Figure 3C**). Similar termination-reinitiation mechanisms also operate in some other families of animal RNA viruses, and in cellular mRNAs (reviewed in Powell, 2010; Gunišová et al., 2017). Interestingly, unlike retroviruses and long terminal repeat (LTR) retrotransposons that translate a dicistonic Gag/CP-Pol/RT RNA via ribosomal frameshifting, non-LTR retrotransposons use a termination-reinitiation mechanism facilitated by the stop-start codon overlap (UAAUG) and a stable hairpin structure located 34 nts downstream of the stop-start (Kojima et al., 2005) (**Figure 3C**). It is unclear, however, how in this particular case eIF3 and the ternary complex are re-acquired by the post-terminating ribosome for efficient reinitiation.

It is worth mentioning that, in CaMV, frameshifting was experimentally ruled out as a mechanism for translation of Gag/CP and Pol/RT in the form of a retrovirus-like Gag-Pol fusion protein (reviewed in Rothnie et al., 1994; Ryabova et al., 2002). Instead, the CaMV ORF V-encoded RT was shown to be expressed separately and its translation appears to be initiated redundantly at one of the two in-frame AUG codons, which are located 35 nts upstream and 7 nts downstream of the CP ORF (ORF IV) stop codon, respectively (Schultze et al., 1990). This suggests that, following termination of ORF IV translation, TAV-mediated reinitiation at an ORF V start codon would require either backward scanning or forward scanning.

It should be emphasized that CaMV TAV-mediated reinitiation after long ORF translation does not appear to depend on any TURBS-like or other *cis*-elements in the viral pgRNA, because TAV can transactivate translation of a second long ORF on the artificial dicistronic RNA lacking any CaMV sequences (Fütterer and Hohn, 1991). Nonetheless, TAV-mediated reinitiation can be facilitated by closeby positions of stop and start codons of the consecutive viral ORFs on pgRNA of CaMV and other plant pararetroviruses that possess TAV (**Figure 2**). Thus, in CaMV (NC_001497), the ORFs I (P1), II (P2) and III (P3) are connected by one nt between the stop and start codons (TAATATG and TAAAATG, respectively), the ORFs III, IV, and V overlap by 19 and 38 nts, respectively, while the ORF VII (P7) and ORF I (MP) are separated by 60 nts; the longer 106 bp spacer between ORF V (RT) and VI (TAV/P6) accommodates core elements (TGACG motif and TATA-box) of the 19S promoter driving Pol II transcription of the subgenomic 19S RNA for translation of the TAV/P6 protein (Driesen et al., 1993) (**Figure 2**, top).

## TAV Activates TOR Kinase to Block Antiviral Autophagy

Another important role for TAV-mediated activation of TOR kinase would be to block cellular autophagy and thereby prevent targeting of viral proteins to autophagosome-mediated degradation (Zvereva et al., 2016; Hafrén et al., 2017a,b). Indeed, CaMV CP and virions are both targeted by antiviral autophagy, and autophagy-deficient Arabidopsis plants are more susceptible to CaMV infection than control wild-type plants (Hafrén et al., 2017a). Importantly, transgenic plants expressing CaMV TAV versions capable of TOR binding and activation are more susceptible to the bacterial pathogen *Pseudomonas syringae* and can even support growth of the *P. syringae* mutant lacking the delivery system for effector proteins required to overcome plant defenses (Zvereva et al., 2016). It was further established that the wild-type TAV/P6, but not its versions incapable of TOR activation, can block oxidative bursts in response to bacterial elicitors of immune responses and interfere with the phytohormone salicylic acid-activated autophagy (Zvereva et al., 2016).

Taken together these findings suggest the TAV-mediated activation of plant TOR kinase is required for both transactivation of viral polycistronic translation and interference with antiviral autophagy (**Figure 4B**). Interestingly, the domain of CaMV TAV responsible for TOR binding and activation (designated dsR, **Figure 4B**) contains a conserved 40 amino-acid RNase H-like motif that binds both RNA-DNA hybrids and double-stranded RNA (dsRNA) *in vitro* (Cerritelli et al., 1998). Based on its dsRNA-binding capacity, this domain was earlier implicated suppression of RNAi triggered by dsRNA. However, the dsR domain turned out to be dispensable for RNAi suppression by TAV/P6 (Zvereva et al., 2016; discussed below).

## Evasion of siRNA-Directed Antiviral Silencing in Plant Pararetroviruses

**FIGURE 5:**
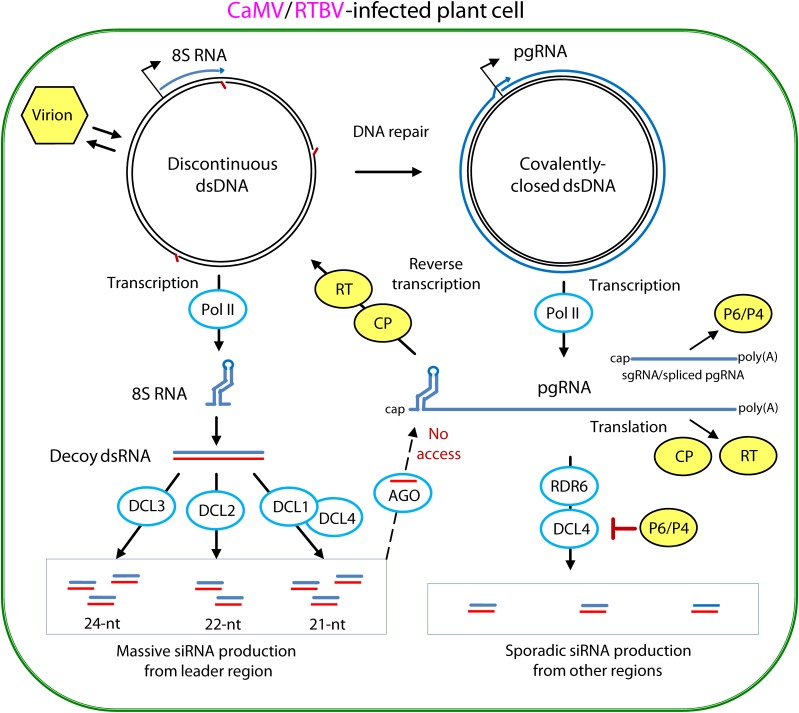
Model for evasion and suppression of RNAi-based antiviral defenses in CaMV- and RTBV-infected cells. Viral DNA is released from the virion into the nucleoplasm. Gaps in the discontinuous dsDNA left after reverse transcription are repaired by the host repair enzymes to create covalently-closed dsDNA. Both repaired and unrepaired forms of viral dsDNA are transcribed by host Pol II. The repaired dsDNA gives rise to viral pgRNA, which serves as a polycistronic mRNA for CP and RT and a template for reverse transcription. In CaMV, Pol II transcription from a separate promoter generates subgenomic 19S RNA, mRNA for TAV/P6, while in RTBV, a fraction of pgRNA is spliced to generate mRNA for P4 protein. Abrupt termination of Pol II transcription at the minus-strand DNA gap (Met-tRNA gap) of the unrepaired dsDNA, results in production of 8S RNA. This RNA forms a viroid-like secondary structure which can be converted by Pol II to dsRNA. The resulting dsRNA serves as a decoy to engage all the four DCLs in massive production of 21-, 22-, and 24-nt viral siRNAs, which then get associates with AGO proteins. Stable secondary structure of the pgRNA leader sequence interferes with complementary interaction of viral siRNA-AGO complexes. The viral CP protein, which initiates packaging of pgRNA in pre-virions and assists its reverse transcription by RT within previrions, can also protect the pgRNA from being targeted by viral siRNAs. The CaMV protein P6 and RTBV P4 interfere with production of viral secondary siRNAs by the RDR6/DCL4 pathways. For further details, please, see the main text. Adapted from Pooggin (2016).

In plant RNAi-based antiviral defense, 21-, 22-, and 24-nt viral small interfering (si)RNAs are generated by four Dicer-like (DCL) enzymes from dsRNA precursors and then sorted by Argonaute (AGO) family proteins to form RNA-induced silencing complexes (RISCs) (Blevins et al., 2006, 2011; Bouché et al., 2006; Fusaro et al., 2006; Aregger et al., 2012; Malpica-López et al., 2018; reviewed in Carbonell and Carrington, 2015; Pooggin, 2016). The 21–22-nt siRNA-RISCs can potentially target viral RNA for cleavage and degradation or translational repression, both causing post-transcriptional gene silencing (PTGS), while 24-nt siRNA-RISCs can potentially target viral DNA for cytosine methylation and transcriptional gene silencing (TGS). Viruses are able to suppress silencing by deploying suppressor proteins targeting different steps of the biogenesis and/or action of viral siRNAs (Csorba et al., 2015) and can potentially evade PTGS and TGS by protecting viral RNA and DNA from RISC access. Plant pararetroviruses transcribing viral circular episomal DNA in the host cell nucleus and then translating and reverse-transcribing pgRNA in the cytoplasm have evolved both suppression and evasion strategies (Pooggin, 2013, 2016; **Figure 5**).

**FIGURE 6:**
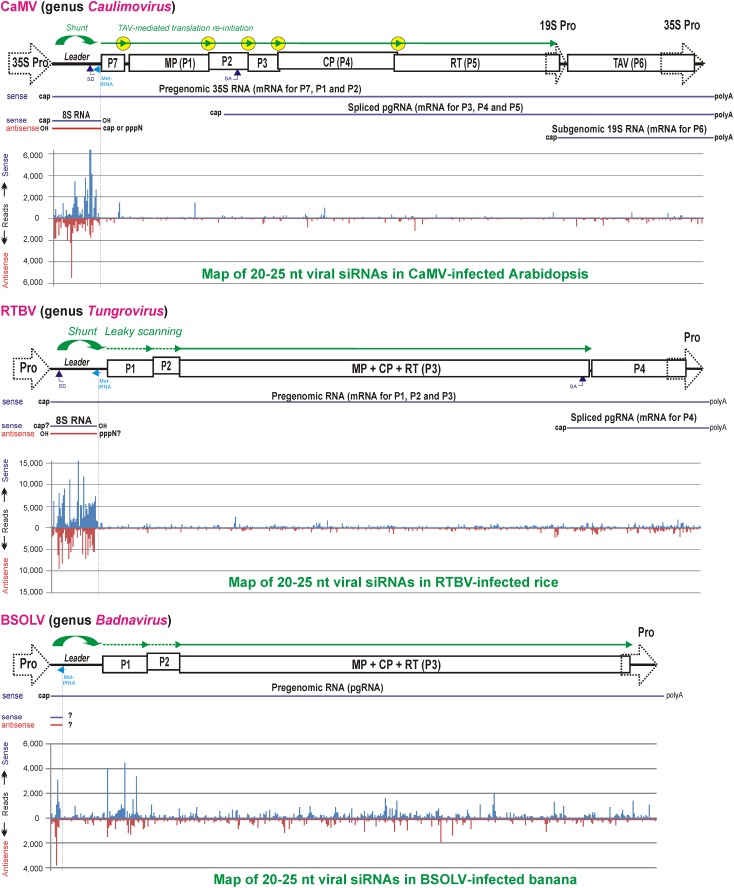
Maps of virus genome-derived siRNAs in the *Caulimovirus* CaMV-infected Arabidopsis, the *Tungrovirus* RTBV-infected rice and the *Badnavirus* BSOLV-infected banana plants. Genome organizations of CaMV, RTBV and BSOLV are shown above the corresponding viral siRNA maps, with promoters, ORFs and polycistronic translation strategies illustrated (see **Figures 2**, **3A,B**). Viral pgRNA, spliced and sgRNAs as well as sense and antisense strands of the decoy 8S dsRNA (see **Figure 5**) are also shown for each virus. On the map histograms, viral 20–25 nt siRNA species mapped on the virus genome in sense and antisense orientation are shown as red and blue bars (with their sizes being proportional to the abundance of each siRNA species). For further details, please, see the main text. Adapted from Blevins et al. (2011), Rajeswaran et al. (2014a,b).

In CaMV and RTBV, the pgRNA leader region plays a role in silencing evasion, owing to its strong secondary structure likely preventing RISC access and also because it encodes a dsRNA decoy engaging all the host DCLs in massive production of 21–24 nt viral siRNAs and thereby protecting other regions of the viral genome from repressive siRNAs (Blevins et al., 2011; Rajeswaran et al., 2014a). Indeed, in CaMV-infected Arabidopsis and RTBV-infected rice plants, viral siRNAs represent a large proportion of total (viral + host) small RNAs (ca. 50 and 19%, respectively), and the 0.6 Kbp leader region is a major hotspot spawning more than 75% of total viral siRNAs derived from the entire 8 Kbp viral genome (**Figure 6**). Illumina sequencing and bioinformatic analysis of viral siRNAs, combined with circularization (c)RT-PCR sequencing of the termini of viral siRNA precursors in Arabidopsis and rice, combined with genetic evidence in Arabidopsis, have allowed to propose a model for viral siRNA biogenesis from the pgRNA leader region (Blevins et al., 2011; Rajeswaran et al., 2014a; Pooggin, 2016). According to this model, two conserved features of the pgRNA leader region, the Met-tRNA primer binding site and the strong, viroid-like, secondary structure, are required for production of the 600 bp dsRNA decoy in the nucleus (**Figure 5**). Viral dsDNA released from virions into the nucleoplasm for Pol II transcription is first repaired by the host DNA repair machinery sealing one or two discontinuities on each strand, the remnants of reverse transcription. If the discontinuity on the DNA minus strand at the Met-tRNA primer binding site is not repaired, Pol II transcription will be terminated at this position and a so-called 8S RNA will be produced. This RNA was first discovered in CaMV-infected turnip plants (Guilley et al., 1982) and then precisely mapped in CaMV-infected Arabidopsis and RTBV-infected rice by cRT-PCR sequencing (Blevins et al., 2011; Rajeswaran et al., 2014a). In both CaMV and RTBV, 8S RNA is 5′-coterminal with pgRNA, terminates at the Met-tRNA binding site and has a complementary RNA (**Figure 6**), presumably produced by an RNA-dependent RNA polymerase activity of Pol II. Since genetic evidence ruled out involvement of the plant silencing-related RNA-dependent RNA (RDR) polymerases 1, 2, and 6 as well as Pol IV and V (Blevins et al., 2011), it is speculated that Pol II would recognize a viroid-like secondary structure of 8S RNA and convert it into the dsRNA decoy for massive production of viral siRNAs (Pooggin, 2016; **Figures 5**, **6**).

It is conceivable that besides CaMV and RTBV the decoy strategy has also been evolved by other plant pararetroviruses with a notable exception of the genus *Badnavirus*, in which the Met-tRNA primer binding site is located at a short distance from the pgRNA transcription start site, in front of the large stem-loop structure (Pooggin et al., 1999; Geering et al., 2005; Rajeswaran et al., 2014b). Indeed, deep small RNA sequencing from banana plants infected with six different badnaviruses revealed that, unlike CaMV and RTBV, viral siRNA production is not restricted to the pgRNA leader region of these badnaviruses (Rajeswaran et al., 2014b). As a consequence of the lack of a decoy-mediated silencing evasion, viral siRNA hotspots are distributed along the viral genome, with abundant siRNAs targeting the protein-coding sequences. Although in some badnaviruses, like, e.g., *Banana streak OL virus* (BSOLV), a short sequence of the pgRNA leader between the transcription and reverse transcription start sites is one of the hotspots for sense and antisense viral siRNAs, a potential short dsRNA precursor of those siRNAs (**Figure 6**; Rajeswaran et al., 2014b) may not serve an effective decoy for sequestering DCLs. It should be noted, however, that, despite production of abundant 21-, 22-, and 24-nt viral siRNAs targeting both non-coding and coding sequences, those six badnaviruses could still persist in vegetative progeny of banana plants for many years and evade cytosine methylation of viral DNA and TGS (Rajeswaran et al., 2014b; reviewed in Pooggin, 2013). This implies other mechanisms evolved by badnaviruses to evade or suppress antiviral PTGS and TGS. In this regard, it is notable that members of the genus *Badnavirus* do not encode any homolog of the *Caulimovirus* TAV/P6 or the *Tungrovirus* P4 which serve as effector proteins suppressing RNAi and other plant defenses in the respective genera of plant pararetroviruses as reviewed below.

Based on a position of the Met-tRNA primer binding site downstream of the leader-based stem-loop structure, the CaMV- and RTBV-type decoy strategy can also be predicted in all members of the genera *Caulimovirus*, *Cavemovirus*, *Petuvirus*, *Soymovirus*, *Rosadnavirus*, and *Orendovirus* as well as in the *Solendovirus* TVCV and the unassigned caulimovirids BFDaV and RuFDV (**Figure 2**, the Met-tRNA binding site highlighted in cyan). In the *Solendovirus* SPVCV and the *Florendovirus* PpersV-sc1, the Met-tRNA binding site is located upstream of the stem-loop structure or on its ascending arm, respectively, suggesting that the run-off transcript would be short, like in the genus *Badnavirus*. Regardless whether or not a dsRNA decoy is expressed, a large and stable stem-loop structure in the pgRNA leader of most plant pararetroviruses is expected to prevent access of the antiviral RISCs charged with viral siRNAs of antisense polarity that can potentially interfere with translation, splicing, packaging or reverse transcription processes regulated by the leader-based *cis*-elements.

## CaMV P6/TAV Suppresses RNAi by Interfering With DCL4-Dependent Amplification of 21-nt siRNAs

Besides its role in suppression of antiviral autophagy and innate immunity responses (Love et al., 2012; Zvereva et al., 2016), the CaMV P6/TAV was found to exert anti-silencing activities (Love et al., 2007; Haas et al., 2008; Shivaprasad et al., 2008). Notably, TAV activity in translation is not required for silencing suppression, because deletion of the TOR-binding domain essential for TAV-mediated transactivation of translation does not influence the ability of CaMV P6 protein to suppress silencing (Zvereva et al., 2016).

The mechanism of CaMV P6-mediated suppression of silencing has been thoroughly investigated (Love et al., 2007; Haas et al., 2008; Shivaprasad et al., 2008; Laird et al., 2013; Zvereva et al., 2016). According to our current model, CaMV P6 interferes with the amplification of 21-nt viral secondary siRNAs mediated by the combined activities of plant RDR6 and DCL4, which reinforces PTGS implemented by RDR6-independent primary siRNAs (Shivaprasad et al., 2008; **Figure 5**). The domain responsible for PTGS suppression likely resides in a C-terminal portion of the CaMV P6 protein (Zvereva et al., 2016) and may not overlap with other functional domains of P6/TAV which are responsible for transactivation of polycistronic translation, suppression of antiviral autophagy and immune responses, and assistance in pgRNA packaging and reverse transcription (**Figure 4B**). It cannot be excluded, however, that a Vir/Avr domain located upstream of the TOR-binding domain (**Figure 4B**; see Kobayashi and Hohn, 2004; references therein), which is highly-variable among CaMV strains, can contribute to suppression of both innate immunity and silencing as was reported by Laird et al. (2013), although a subsequent study did not confirm Vir/Avr involvement in silencing suppression (Zvereva et al., 2016).

It should be emphasized that CaMV P6/TAV is an abundant viral protein available for interaction with its multiple cellular and viral partners in the cytoplasm where it holds together the viral replication factories (inclusion bodies or viroplasm) and serves in viral pgRNA translation and reverse transcription, viral protein stabilization, virion assembly, movement and transmission (Shepherd et al., 1980; Covey and Hull, 1981; Pfeiffer and Hohn, 1983; Modjtahedi et al., 1984; Himmelbach et al., 1996; Kobayashi et al., 1998; Lutz et al., 2012; Angel et al., 2013; Bak et al., 2013; Geldreich et al., 2017; reviewed in Hohn, 2013; Hohn and Rothnie, 2013; Schoelz and Leisner, 2017), as well as in suppression of antiviral defenses (autophagy, innate immunity and PTGS; as discussed above). In addition, CaMV P6 is visiting the nucleus (Haas et al., 2005, 2008) where it may assist in the nuclear export of unspliced viral pgRNA and interfere with TGS of CaMV minichromosomes (Al-Kaff et al., 1998; Pooggin, 2013). A nuclear role for P6/TAV in suppression of DCL4-mediated PTGS was also proposed, in which a nuclear P6 targets a dsRNA-binding protein DRB4, the partner of DCL4 (Haas et al., 2008). However, such a nuclear-localized interaction of CaMV P6 with a DRB4/DCL4 complex may not be compatible with the main cytoplasmic function of DCL4 in processing RDR6-dependent dsRNAs derived from miRNA-targeted transcripts of endogenous tasiRNA genes (Rajeswaran et al., 2012) and dsRNA replicative intermediates of cytoplasmic RNA viruses (Bouché et al., 2006; Fusaro et al., 2006; Malpica-López et al., 2018; reviewed in Pooggin, 2016). It cannot be formally excluded that the mutations in the two nuclear localization signals (NLS1 and NLS2; **Figure 4B**), which resulted in exclusively cytoplasmic localization of CaMV P6/TAV and abolished its negative effect on DCL4-dependent tasiRNA biogenesis (Haas et al., 2008), could have affected a potential interaction of P6 with an unidentified component of the cytoplasmic RNAi machinery. Based on dispensability of both Vir/Avr and dsR domains for CaMV P6-mediated interference with DCL4 processing of tasiRNA precursors (Zvereva et al., 2016), we speculate that a silencing suppression domain is located in a C-terminal portion of the P6 protein which contains NLS2, an RNA-binding domain “b” (RBb, also involved in interaction with the viral CP), and a Zn-finger domain (Zn) of unknown function (**Figure 4B**). The nuclear export signal (NES) and a P6–P6 interaction domain located at the N-terminus of CaMV P6 (Haas et al., 2005; **Figure 4B**) may also contribute to P6-mediated suppression of silencing in the cytoplasm.

## RTBV P4 May Suppress Antiviral RNAi and Innate Immunity

The tungrovirus RTBV possesses a unique protein P4, which is not present in other genera of *Caulimoviridae* (with possible exception for the genus *Orendovirus* containing ancient, RTBV-like pararetroviruses integrated and decayed in the rice genome as discussed above; **Figure 2**). RTBV P4 is translated from a monocistronic mRNA, generated by splicing of the pgRNA (Fütterer et al., 1994), and does not exhibit any translational transactivator activity, which is consistent with the leaky scanning strategy of polycistronic translation of RTBV pgRNA (Fütterer et al., 1997; **Figure 3B**). Recently, it was discovered that RTBV P4 can potentially interfere with spread of RNA silencing mediated by DCL4-dependent mobile 21-nt siRNAs (Rajeswaran et al., 2014a). Using a classical transient silencing suppression assay in leaves of *Nicotiana benthamiana* GFP-transgenic line 16c, RTBV P4 was found to block cell-to-cell movement of GFP transgene silencing and simultaneously enhance cell-autonomous GFP silencing, which well correlated with dramatic decrease in accumulation of mobile 21-nt GFP siRNAs and concomitant increase in accumulation of 22-nt GFP siRNAs. Thus, similar to CaMV P6, RTBV P4 appears to interfere with the biogenesis of 21-nt siRNAs likely mediated by DCL4 (Rajeswaran et al., 2014a), the primary antiviral Dicer involved in defense against both RNA and DNA viruses including CaMV (Blevins et al., 2006, 2011). In the *N. benthamiana* GFP silencing system, the dsRNA precursors presumably produced by RDR6 and then processed by DCL4 into the 21-nt mobile siRNAs responsible for limited cell-to-cell movement of GFP silencing (Himber et al., 2003), become available for DCL2 generating 22-nt siRNAs when RTBV P4 is expressed: those 22-nt siRNAs are likely responsible for the enhanced cell-autonomous silencing (Rajeswaran et al., 2014a). It remains to be investigated if P4 interferes with cell-to-cell spread of antiviral silencing in RTBV-infected rice plants. The mechanism of P4-mediated suppression of antiviral defenses remains to be further investigated. In particular, it would be interesting to explore if RTBV P4 can also suppress innate immunity and TOR-dependent autophagy, similar to CaMV P6/TAV.

## Concluding Remarks and Evolutionary Implications for the Family *Caulimoviridae*

The evolution of a ribosome shunt configuration in the pgRNA leader region of plant pararetroviruses appears to have been driven by the necessities to regulate sorting of pgRNA for translation and reverse transcription and to protect the pgRNA leader sequence from the repressive action of virus-derived siRNAs generated by the plant RNAi-based antiviral defense system. The preservation of shunt configuration in all genera of the family *Caulimoviridae*, including ancient florendoviruses integrated in the genomes of many flowering plants, argues for appearance of an original shunt configuration in a progenitor plant pararetrovirus, possibly through an inverted repeat in its non-coding region. Both primary sequence and secondary structure elements in the pgRNA leader have then evolved to regulate (fine-tune) translation, packaging and reverse transcription of pgRNA as well as its transcription, polyadenylation and splicing. In genera *Tungrovirus* and *Caulimovirus*, positioning of the RT primer binding site close to the end of the pgRNA leader, combined with a stable viroid-like secondary structure of 8S RNA, the run-off transcript terminating at the primer binding site and 5′-coterminal with pgRNA, has enabled to express a dsRNA decoy engaging the RNAi machinery in massive siRNA production and thereby strongly reducing siRNA production from other regions of the viral genome. Other genera of *Caulimoviridae* with the RT binding site located downstream of the structured leader region may have evolved a similar decoy strategy of silencing evasion. Another milestone in the evolution of plant pararetroviruses was the acquisition of a unique gene encoding the transactivator protein TAV, which enabled to translate two and more consecutive large viral ORFs from a single pgRNA or its spliced variant. This acquisition has allowed several genera of *Caulimoviridae* to split a large ORF encoding MP, CP, and RT in the form of a polyprotein into separate ORFs, which would enable regulated expression of these “core” viral proteins, and to acquire other accessory proteins as separate ORFs (**Figure 2**). With the exception of genus *Petuvirus* possessing only one large ORF, those genera that lack TAV have also found a way to acquire accessory protein-coding ORFs, upstream and/or downstream of the MP-CP-RT polyprotein ORF. In the former case (e.g., in genera *Tungrovirus* and *Badnavirus*), the leaky scanning strategy was developed to clear the accessory ORFs of internal AUGs and thereby allow fractions of the scanning ribosomes to initiate translation at the start codons of three consecutive ORFs on the polycistronic pgRNA. In the genus *Tungrovirus*, the accessory ORF location downstream of the MP-CP-RT ORF forced to evolve splicing of pgRNA. Interestingly, the accessory protein P4 encoded by this ORF in RTBV is implicated in suppression of plant RNAi- and innate immunity-based antiviral defenses. Likewise, the TAV protein, in addition to its function in viral polycistronic translation, plays a role in suppression of antiviral defenses as established for the *Caulimovirus* CaMV. Notably, TAV-mediated activation of the plant protein kinase TOR promotes reinitiation of translation after long ORFs on the viral pgRNA and simultaneously blocks antiviral autophagy and innate immunity responses, while interaction of TAV with component(s) of the plant RNAi machinery interferes with antiviral silencing. It is conceivable that functional interactions of the CaMV TAV with multiple host proteins promoting viral polycistronic translation and counter-defense as well as with viral proteins involved in viral replication and movement (**Figure 4B**) are conserved in other members of genus *Caulimovirus* as well as other genera of *Caulimoviridae* encoding TAV homologs. It is also conceivable that, in those genera of *Caulimoviridae* that do not possess TAV, other accessory protein(s) have evolved to actively suppress plant antiviral defenses, as exemplified by the case of *Tungrovirus* protein P4.

## Author Contributions

MP and LR contributed to writing and editing the manuscript. MP performed the conceptual and graphical design and description in the main text of all aspects of the models presented in **Figures 1**, **2**, **3A,B**, **4B**, **5**, **6**. LR performed the conceptual and graphical design and description in the main text of all aspects of the models presented in **Figures 4A**, **3C,D**. MP and LR approved the submitted manuscript.

## Conflict of Interest Statement

The authors declare that the research was conducted in the absence of any commercial or financial relationships that could be construed as a potential conflict of interest. The handling Editor declared a shared affiliation, though no other collaboration, with one of the authors LR.

## Footnotes

1 https://talk.ictvonline.org/taxonomy

## Abbreviations

AGO: Argonaute
BFDaV: *Blueberry fruit drop-associated virus*
BSOLV: *Banana streak OL virus*
CaMV: *Cauliflower mosaic virus*
CP: coat protein
cRT-PCR: circularization RT-PCR
DCL: Dicer-like
DRB: dsRNA-binding
dsDNA: double-stranded DNA
dsRNA: double-stranded RNA
eIF: eukaryotic translation initiation factor
eL13: eukaryotic 60S ribosomal protein L13
eL18: eukaryotic 60S ribosomal protein L18
eL24: eukaryotic 60S ribosomal protein L24
FMV: *Figwort mosaic virus*
MP: movement protein
NES: nuclear export signal
NLS: nuclear localization signal
nt: nucleotide
nts: nucleotides
PFV: *Prototype foamy virus*
pgRNA: pregenomic RNA
Pol: polymerase
PpersV: *Prunus persica virus*
PTGS: post-transcriptional gene silencing
RB: RNA-binding
RDR: RNA-dependent RNA polymerase
RISP: reinitiation supporting protein
RNAi: RNA interference
rRNA: ribosomal RNA
RT: reverse transcriptase
RTBV: *Rice tungro bacilliform virus*
RTSV: *Rice tungro spherical virus*
RuFDV: *Rudbeckia flower distortion virus*
RYVV: *Rose yellow vein virus*
S6K1: ribosomal protein S6 kinase 1
SA: splice acceptor
SD: splice donor
sgRNA: subgenomic RNA
siRNA: small interfering RNA SPPV *Sweet potato pakakuy virus*
tasiRNA: *trans*-acting siRNA
TAV: transactivator/viroplasmin
TGS: transcriptional gene silencing
TOR: target of rapamycin
tRNA: transfer RNA
TURBS: termination upstream ribosome binding site
uORF: upstream open reading frame
Vir/Avr: virulence/ avirulence
